# Association between indwelling urinary catheters and the poor prognosis among elderly patients with pulmonary infection

**DOI:** 10.3389/fcimb.2026.1873862

**Published:** 2026-07-30

**Authors:** Jiewei Cui, Yuanyuan Cui, Qian Li, Chunyan Li, Miaoyu Wang, Zhixin Liang

**Affiliations:** 1 Senior Department of Pulmonary & Critical Care Medicine, Chinese PLA General Hospital, Beijing, China; 2 Shandong People’s Armed Police Hospital, Jinan, China; 3 Department of Pulmonary and Critical Care Medicine, First Medical Center of Chinese PLA General Hospital, Beijing, China

**Keywords:** association, elderly, indwelling urinary catheterization, mortality, pulmonary infection, risk factor, urinary tract infection (UTI)

## Abstract

**Objective:**

With the rapid aging of society, the number of elderly patients with pulmonary infection (PI) is increasing significantly. Indwelling urinary catheters (IUC) are commonly used in elderly patients and appear to be associated with poor prognosis in this population; however, supporting evidence remains insufficient. Therefore, this study aimed to clarify the association between IUC and 30-day mortality in elderly patients with PI.

**Methods:**

This retrospective study was conducted at one of the largest tertiary general hospitals in China. We enrolled all elderly patients with PI who did or did not undergo IUC, and statistically analyzed the association mentioned above.

**Results:**

By retrospectively searching the hospital’s patient medical database, a total of 700 patients (aged ≥65 years) with PI were screened from January 1, 2018, to December 31, 2023, with an overall 30-day mortality of 17.3% (121/700). Among these patients, 295 (42.1%, 295/700) had an IUC within 72 hours of admission. Compared with patients without IUC, patients with IUC had a higher proportion of urinary tract infection (18.9% vs. 9.7%), female gender (35.3% vs. 25.2%), CURB-65 score ≥2 (73.7% vs. 37.2%), residence in the respiratory intensive care unit (RICU admission) (49.2% vs. 21.7%), and 30-day mortality (28.1% vs. 9.4%). Kaplan-Meier (KM) survival curves demonstrated that IUC, rather than urinary tract infection, was significantly associated with 30-day mortality (P<0.001). Cox multivariate regression analysis revealed that IUC was an independent risk factor for 30-day mortality compared with urinary tract infection (hazard ratio [HR]=2.8, 95% confidence interval [CI] 1.9-4.2, P<0.001)). However, after adjusting for four confounding factors (urinary tract infection, CURB-65 score ≥2, RICU admission, female gender and mechanical ventilation), IUC was no longer an independent risk factor, though it still increased the 30-day mortality by 30% (HR = 1.3, 95% CI: 0.8–2.0, P = 0.246).

**Conclusion:**

Indwelling urinary catheterization, rather than urinary tract infection, was associated with 30-day mortality in elderly patients with pulmonary infection. Indwelling urinary catheter is a double-edged sword: while it is an important medical tool, it is by no means harmless. The principle of “an unnecessary urinary catheter is also harmful” should be strictly incorporated into clinical guidelines for indwelling urinary catheters.

## Introduction

According to the Global Burden of Disease (GBD) 2021 database, lower respiratory tract infections, predominantly manifested as pulmonary infection (PI), remain one of the most prevalent and fatal infectious diseases worldwide, with their incidence and mortality rising markedly with age. Amid progressive global population aging, the disease burden of elderly patients with PI has increased substantially. Specifically, the number of lower respiratory tract infection cases in individuals aged over 70 years increased from 45.8 million in 1990 to 100.0 million in 2019, with the corresponding death toll rising from 218,000 to 314,000 over the same period (Infections GBDLR and Antimicrobial Resistance C, 2024). Consequently, the burden of prevention and treatment of elderly PI has increasingly significant implications for healthcare systems worldwide (Liao et al., 2025; Wang et al., 2025).

Similarly, data from the Global Burden of Disease Study 2021 (GBD 2021) confirmed that the global disease burden of urinary tract infection (UTI) has also been continuously escalating over the past three decades (He et al., 2025). Driven globally by population aging and rising bacterial antimicrobial resistance, the global total number of UTI cases surged from 2.698 billion to 4.491 billion between 1990 and 2021, representing a 66.45% relative increase. UTI disproportionately affects older populations, with advanced age serving as a consistent risk factor for higher UTI incidence. A prospective observational study further demonstrated that the two-year cumulative incidence of UTI was significantly higher in elderly patients aged ≥65 years (38.0%) compared with younger counterparts (12.8%), even among individuals without IUC (Al Qahtani et al., 2024). The higher susceptibility in older adults is partially attributable to age-related comorbidities that frequently require indwelling urinary catheterization (IUC), including benign prostatic hyperplasia and long-term bedridden paralysis (Kim et al., 2017; Wakefield, 2021). Notably, IUC confers a substantial risk of healthcare-associated UTI: the infection risk increases by approximately 3%–10% per catheterization day and approaches 100% within 30 days of placement (Clawson et al., 2022; Venkataraman and Yadav, 2023).

Given the high prevalence of both PI and UTI in elderly populations, UTI has been recognized as the second most common infectious diseases requiring hospitalization among adults aged ≥65 years, second only to pulmonary infection, thereby imposing a substantial socioeconomic and clinical burden on the elderly (Cortes-Penfield et al., 2017; Esme et al., 2019; Yuruk Atasoy et al., 2026). Nevertheless, comprehensive epidemiological analyses based on the latest GBD 2021 data focusing on this dual infectious burden remain insufficient (Hua et al., 2021).

As frontline clinicians specializing in respiratory medicine, our clinical practice routinely involves the diagnosis and management of elderly patients with PI and UTI. Consistent clinical observations indicate that elderly pneumonia patients with concurrent UTI tend to exhibit more severe clinical manifestations and poorer long-term prognoses. Accumulated evidence has demonstrated that UTI not only prolongs hospital stay and increases medical costs but also increases all-cause mortality across multiple diseases (Ovbiagele et al., 2006; Monaghan et al., 2011; Glassou et al., 2017). Several studies have further suggested that superimposed UTI may exacerbate mortality risk in patients with preexisting pulmonary infection (Leangpanich et al., 2019; Alemu et al., 2020). However, existing evidence remains controversial and inconclusive. One previous study reported discrepant findings: although univariate analysis showed higher 30-day mortality in PI patients with concurrent UTI, multivariate regression revealed that UTI was not an independent prognostic factor for 30-day mortality in patients with nursing and healthcare-associated pneumonia (P = 0.17) (Yamazoe et al., 2019).

Despite these conflicting findings regarding the prognostic impact of UTI on PI patients, our real-world clinical observations suggest that elderly PI patients with IUC, particularly those with indwelling urinary catheterization, consistently present worse clinical outcomes and higher mortality. However, the real-world evidence quantifying their independent prognostic effects remains scarce, leaving an unmet clinical gap. Accordingly, the present study aimed to clarify the prognostic roles of indwelling urinary catheters and concurrent UTI in elderly patients with pulmonary infection (PI), and further quantify their correlation with poor clinical prognoses.

## Materials and methods

### Study design and patient source

The General Hospital of the Chinese People’s Liberation Army is one of the largest comprehensive tertiary care centers in China. To investigate the association between IUC and poor prognosis in elderly patients with PI, this retrospective study retrieved clinical data from the hospital’s electronic medical record database, enrolling all elderly patients aged ≥65 years diagnosed with PI admitted to the Department of Pulmonary and Critical Care Medicine.

### Patient screening and eligibility criteria

#### Search terms and inclusion criteria

Eligible patients were those aged ≥65 years who were hospitalized in the Department of Pulmonary and Critical Care Medicine between January 1, 2018, and December 31, 2023, with a diagnosis of PI. Patients were identified by searching the hospital’s medical database for PI and related diagnoses established after admission. Search terms for the primary post-admission diagnosis included: “pulmonary infection”, “pneumonia”, “community-acquired pneumonia”, “bacterial pneumonia”, “fungal pneumonia”, “mycoplasma pneumonia”, “chlamydial pneumonia”, and “viral pneumonia”.

#### Patients exclusion criteria

Patients were excluded if they met any of the following criteria: (1) Initially diagnosed with PI after admission but reclassified as having non-infectious pulmonary diseases (e.g., pulmonary embolism, interstitial pneumonia, lung cancer) upon discharge; (2) Transferred to other departments (e.g., cardiology, surgery) within 30 days of admission; (3) Had multiple consecutive hospitalizations (only data from the first hospitalization were included to exclude hospital-acquired pneumonia); (4) Underwent IUC more than 72 hours after admission (to ensure valid grouping based on early post-admission catheterization status); (5) Were diagnosed with human immunodeficiency virus (HIV) infection (no such cases were identified in this study); (6) Had missing key clinical data.

#### Definition of urinary tract infection

First, we detailed our universal urinary screening strategy for hospitalized patients. Given the ultra-low cost and excellent cost-effectiveness of routine urinalysis (including urinary leukocytes, erythrocytes, and other inflammatory indicators), all patients admitted to the Respiratory Medicine ward and the Respiratory Intensive Care Unit (RICU) receive standardized routine urinalysis within 24 hours of admission. This universal admission screening enables early and comprehensive detection of potential urinary system disease and subclinical urinary inflammation, ensuring a complete baseline urinary evaluation for all enrolled elderly patients with PI, including those with IUC. Notably, the urinary leukocyte detection method adopted in our hospital is based on a neutrophil esterase assay, and the leukocytes identified via this routine testing are predominantly neutrophils, which specifically reflect urinary inflammatory responses. The grading of urinary tract infections through neutrophil esterase testing and the corresponding estimated urine leukocyte count by flow cytometry were as follows: (-) about <15/μl; (±) about 15–75/μl; (1+) about 75–125/μl; (2+) about 125–500/μl; and (3+) about >500/μl.

In our standardized clinical practice, isolated mild neutrophil elevation with a leukocyte grade below 2+ is generally considered to represent either the absence of true UTI or only trivial, clinically insignificant urinary inflammation. Such mild urinary abnormalities exert no substantial impact on the antimicrobial treatment strategy or short-term prognosis of elderly patients with PI, and thus are not specifically monitored or intervened in routine clinical practice. Accordingly, we do not perform routine urine culture for these mild urinary abnormalities to avoid redundant microbial testing and unnecessary medical costs. In contrast, we implement a unified and rigorous threshold for urine culture in catheterized patients: urine culture is routinely performed only when urinary neutrophil leukocyturia reaches 2+ or above. Due to the high specificity of neutrophil esterase for urinary infection, a leukocyte grade of 2+ or higher strongly indicates clinically significant UTI. This diagnostic criterion is particularly reliable for our study population of elderly patients with PI (≥65 years), who have inherently higher susceptibility to urinary tract complications. Therefore, this threshold ensures accurate and consistent UTI ascertainment for our prognostic analysis. However, consistent with real-world clinical practice, the overall positive rate of urine culture remains relatively low under this standardized screening strategy.

### Collection of clinical data

Clinical data collected within 72 hours of admission included: IUC status, IUC indications, the grading urinary leukocyte count (-/±/1+/2+/3+), and potential prognostic factors for PI, such as age, female gender, disease severity (assessed via CURB-65 score), comorbidities (cardiovascular and cerebrovascular diseases, diabetes mellitus, chronic renal failure, structural lung diseases including bronchiectasis, mechanical ventilation (MV), chronic obstructive pulmonary disease, and emphysema), treatment history within 90 days prior to admission (hospitalization for other conditions, use of broad-spectrum antibiotics, use of immunosuppressants), current hospitalization symptoms, admission to the Respiratory Intensive Care Unit (RICU), appropriateness of empirical antimicrobial therapy, and infection-related inflammatory biomarkers (peripheral blood WBC count).

Prognostic data included 30-day survival status (survival or death) after admission. For patients discharged within 30 days, 30-day outcomes were confirmed via telephone follow-up.

### Statistical analysis

Continuous variables were described as mean (standard deviation, SD) or median (interquartile range, IQR) based on their compliance with normal distribution requirements. Categorical variables were presented as frequencies and percentages (n, %). Kaplan-Meier (KM) curves were utilized to illustrate differences in 30-day survival among patient groups stratified by indwelling urinary catheter (IUC) placement and urinary tract infection (UTI) status. Cox proportional hazards regression analysis was performed to assess whether IUC and UTI were risk factors for 30-day mortality in elderly patients with PI. Statistical results were expressed as odds ratios (OR) or hazard ratios (HR) along with their corresponding 95% confidence intervals (95% CI). A two-tailed P-value < 0.05 was considered statistically significant.

## Results

### Baseline characteristics of enrolled patients

As presented in **Table 1**, a total of 760 elderly patients with PI were admitted to the Department of Pulmonary and Critical Care Medicine between 2018 and 2023. Owing to the retrospective nature of the study, complete clinical data were not obtainable for all patients: 38 patients were excluded due to missing records on indwelling urinary catheter (IUC) placement status within 72 hours of admission, and an additional 22 patients were excluded because their 30-day survival outcomes (alive or deceased) could not be confirmed. Ultimately, 60 patients were excluded (exclusion rate: 7.9%, 60/760), leaving 700 elderly PI patients for the final analysis.

**Table 1:** Population baseline characteristics of all enrolled elderly patients with pulmonary infection with and without indwelling urinary catheters (IUCs).

(N) Mean (SD)/N(%)	Total 700 (100%)	Without IUC 405 (57.9%)	With IUC 295 (42.1%)	P-value
Age (years)	83.9 (7.7)	83.5 (7.8)	84.4 (7.6)	0.169
>=65, <75	100 (14.3%)	62 (15.3%)	38 (12.9%)	0.608
>=75, <85	204 (29.1%)	119 (29.4%)	85 (28.8%)	
>=85	396 (56.6%)	224 (55.3%)	172 (58.3%)	
Urinary tract infections				**<0.001**
No	500 (71.4%)	337(83.2%)	163(55.3%)	
Yes	200 (28.6%)	68(16.8%)	132(44.7%)	
Outcome within 30 days				**<0.001**
Survival	579 (82.7%)	367 (90.6%)	212 (71.9%)	
Non-survival	121 (17.3%)	38 (9.4%)	83 (28.1%)	
CURB-65 score				**<0.001**
0 and 1 score	341 (48.7%)	256 (63.2%)	85 (28.8%)	
2 to 5 score	359 (51.3%)	149 (37.2%)	210 (73.7%)	
Gender				**0.004**
Male	494 (70.6%)	303 (74.8%)	191 (64.7%)	
Female	206 (29.4%)	102 (25.2%)	104 (35.3%)	
RICU admission	233 (33.3%)	88 (21.7%)	145 (49.2%)	**<0.001**
**Comorbidities**	516 (73.7%)	294 (72.6%)	222 (75.3%)	0.43
Diabetes	182 (26.0%)	104 (25.7%)	78 (26.4%)	0.821
Tumor	71 (10.1%)	48 (11.9%)	23 (7.8%)	0.079
Cardiovascular and cerebrovascular diseases	520 (74.3%)	304 (75.1%)	216 (73.2%)	0.582
Chronic renal failure	92 (13.1%)	49 (12.1%)	43 (14.6%)	0.338
Structural lung diseases	189 (27.0%)	117 (28.9%)	72 (24.4%)	0.187
Mechanical ventilation	112 (16.0%)	30 (7.4%)	82 (27.8%)	**<0.001**
Treatments within 90 days prior to this hospitalization				
Hospitalization history due to other diseases	86 (12.3%)	49 (12.1%)	37 (12.5%)	0.86
History of immunosuppressive drugs	51 (7.3%)	31 (7.7%)	20 (6.8%)	0.66
History of broad-spectrum antibacterial drugs	86 (12.3%)	43 (10.6%)	43 (14.6%)	0.115
Main symptoms of infection				
Fever>37.3 °C	462 (66.0%)	274 (67.7%)	188 (63.7%)	0.279

RICU, Respiratory Critical Care Unit.

Values in bold indicate statistically significant differences (P < 0.05).

All enrolled patients were aged ≥65 years, with a male predominance (70.6%, 494/700), and fever was the primary presenting symptom of PI. The overall 30-day mortality rate among the study cohort was 17.3% (121/700). Of the 700 patients, 295 (42.1%) underwent IUC within 72 hours of admission.

Compared with patients without IUCs, those with IUCs exhibited a higher prevalence of several clinical characteristics, including urinary tract infection (UTI) (18.9% vs. 9.7%), female gender (35.3% vs. 25.2%), CURB-65 score >2 (73.7% vs. 37.2%), admission to the Respiratory Intensive Care Unit (RICU) (49.2% vs. 21.7%), and 30-day mortality (28.1% vs. 9.4%). Most patients had multiple comorbidities, which was likely attributed to their advanced age; however, the distribution of comorbidity prevalence did not differ significantly between the IUC and non-IUC groups.

Supplementary data on IUC indications: A total of 295 patients received indwelling urinary catheterization in this study. Due to the inherent limitations of retrospective real-world research, complete and accurate records of IUC insertion reasons were not fully available in electronic medical records. Specifically, 48 of the 295 catheterized patients had no clear documented catheterization indications and could not be accurately classified. For the remaining 247 patients with retrievable clinical records, we categorized the specific IUC insertion indications as follows: acute and chronic urinary retention (153 cases, 62.0%), critical illness monitoring due to severe infection-induced altered mental status (68 cases, 27.5%), severe incontinence combined with prolonged bedridden status and pressure injury risk (16 cases, 6.5%), age-related voiding dysfunction (7 cases, 2.8%), benign prostatic hyperplasia (2 cases, 0.8%), and neurological dysfunction (1 case, 0.4%).

### Survival analysis of IUC and UTI in elderly patients with pulmonary infection

As presented in **Table 1**, elderly patients with PI who had IUC (IUCs) exhibited significantly higher 30-day mortality compared to those without IUCs (P < 0.05). However, the association between urinary tract infection (UTI) and mortality in this elderly PI population remained unclear.

**Figure 1:**
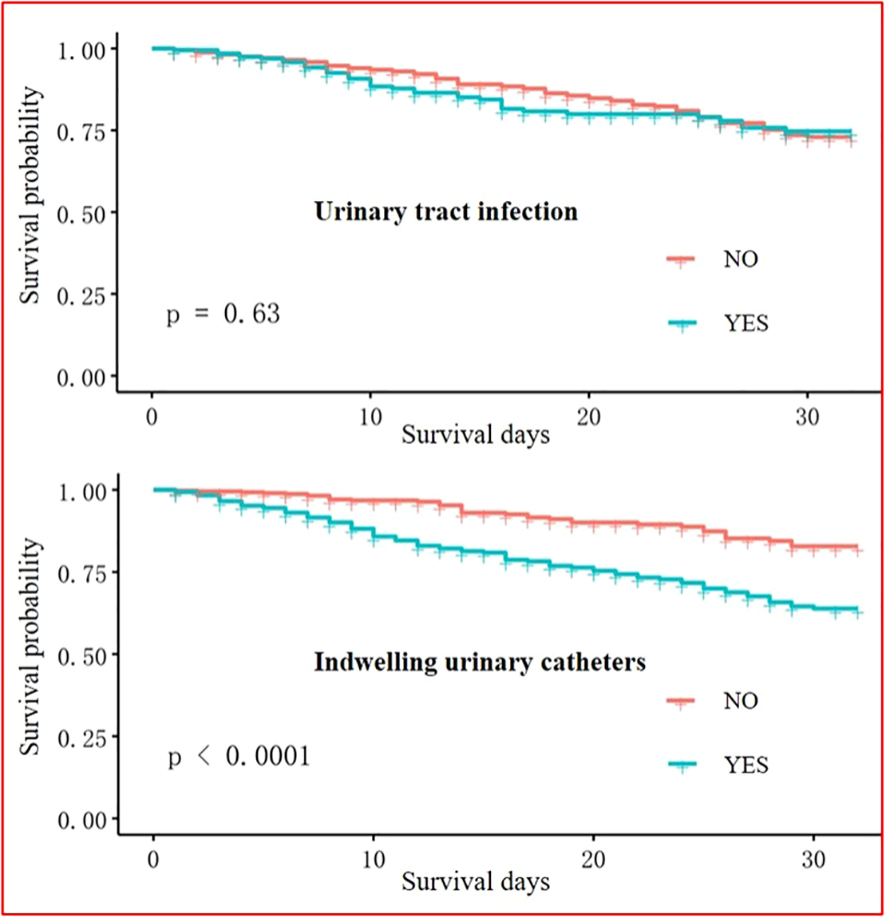
The Kaplan-Meier (KM) survival curves of indwelling urinary catheters (IUCs) and urinary tract infections (UTIs) in elderly patients with pulmonary infection. The results showed that IUCs placement were associated with a marked increase in 30-day mortality than those without IUCs placement in elderly patients with pulmonary infection (P<0.0001). But there was no significant difference in 30-day mortality between those patients with and without UTIs (P = 0.63).

To address this uncertainty, Kaplan-Meier (KM) survival curves were generated to explore whether UTI—similar to IUC—was correlated with 30-day mortality in elderly PI patients. As illustrated in **Figure 1**, the results confirmed that IUC was associated with a marked increase in 30-day mortality (P<0.001). Notably, however, there was no significant difference in 30-day mortality between elderly PI patients with and without UTIs ((P = 0.63). These findings indicate that 30-day mortality in elderly individuals with pulmonary infection is unrelated to UTI status.

### Cox regression analysis of IUC and 30-day mortality in elderly patients with pulmonary infection

Cox regression analysis was performed to clarify two key questions: whether indwelling urinary catheter (IUC) placement was an independent risk factor for 30-day mortality in elderly patients with PI, or if its association with increased mortality was merely a reflection of other confounding clinical risk factors.

Univariate Cox regression results (**Table 2**) identified IUC as a risk factor for 30-day mortality in elderly PI patients: those with IUCs had a 2.7-fold higher risk of 30-day mortality compared to those without IUCs (HR = 2.7, 95% CI: 1.8–3.9, P < 0.001).

**Table 2:** Cox multiple regression analysis about the association between indwelling urinary catheters (IUC) and the 30-day mortality among elderly patients with pulmonary infection.

	Survival outcome - HR (95%CI) P-value			
	Non-adjusted	Model 1*	Model 2#	Model 3†
**IUC**	2.7 (1.8, 3.9) <0.001	2.8 (1.9, 4.2) <0.001	1.4 (0.9, 2.1) 0.149	1.3 (0.8, 2.0) 0.246
Survival days of dead elderly patients with IUC			(83) 13.3 (8.4)	(N) Mean (SD)
Survival days of dead elderly patients without IUC			(38) 15.1 (8.0)	
Survival days of all dead elderly patients			(121) 13.9 (8.2)	

Time variable: Survival days of dead elderly patients with pulmonary infection;

Exposure variable: Indwelling urinary catheters (IUC);

Outcome variables: Survival within 30 days or not (30-day mortality);

Model 1*: Adjust for: Urinary tract infections (UTI solely);

Model 2#: Adjust for: UTI, RICU admission, gender (Female), CURB65 score (0–1 vs 2-5);

Model 3†: Adjust for: UTI, RICU admission, gender (Female), CURB65 score (0–1 vs 2-5) and Mechanical ventilation (MV).

Values in bold indicate statistically significant differences (P < 0.05).

When urinary tract infection (UTI) was included as an adjustment variable in the multivariate Cox regression model (Model 1), IUC remained an independent risk factor for 30-day mortality (HR = 2.8, 95% CI: 1.9–4.2, P < 0.001). However, after further adjusting for four additional mortality-related variables (CURB-65 score > 2, female gender, comorbidities, and RICU admission) (Model 2), IUC no longer qualified as an independent risk factor for 30-day mortality, despite a 40% increase in mortality risk (HR = 1.4, 95% CI: 0.9–2.1, P = 0.149).

In the initial data extraction phase, mechanical ventilation (MV) status was not collected because of strong collinearity between MV and RICU admission; ventilated patients have far higher RICU admission rates, and adding MV into the adjustment model would further attenuate the HR of IUC without changing core conclusions (no significant difference, P>0.05). To enhance data integrity, we retrospectively supplemented mechanical ventilation data for all 700 enrolled patients and completed supplementary Cox regression analysis incorporating MV as an additional covariate. After adding mechanical ventilation into the fully adjusted model (Model 3 in **Table 2**), the predictive effect of IUC on 30-day mortality was further weakened (HR = 1.3, 95% CI: 0.8–2.0, P = 0.246), further verifying that IUC was not an independent risk factor for poor prognosis after fully adjusting for multiple disease severity-related confounders. The HR of IUC in Model 3 remained above 1, suggesting a mild adverse prognostic trend without statistical significance.

## Discussion

This single-center retrospective cohort study enrolled 700 elderly patients aged ≥65 years with pulmonary infection (PI) to systematically explore the distinct prognostic effects of indwelling urinary catheters (IUC) and concurrent urinary tract infection (UTI) on 30-day mortality in this vulnerable population. Baseline characteristic analysis revealed that patients with early IUC presented significantly higher burdens of adverse clinical factors, including higher UTI incidence, female predominance, CURB-65 score ≥2, increased RICU admission rate, increased mechanical ventilation rate and higher 30-day mortality, compared with non-IUC patients. Subsequent Kaplan-Meier survival analysis further clarified that poor short-term survival was specifically associated with IUC rather than UTI complication. Consistent with the survival trend, univariate Cox regression identified IUC as a significant mortality risk factor for elderly patients with pulmonary infection. Notably, IUC remained an independent prognostic risk factor after adjustment for UTI alone. However, after comprehensive adjustment for multiple key confounding variables covering disease severity and baseline clinical differences (UTI, CURB-65 score ≥2, RICU admission, and female gender), IUC lost its independent predictive association with 30-day mortality. Importantly, supplementary Cox regression analysis incorporating mechanical ventilation as an additional disease severity covariate further verified the robustness of our conclusions.

Against the backdrop of global population aging acceleration, the hospitalization rate and disease burden of elderly patients with PI have continued to rise worldwide. Elderly individuals often present with multiple comorbidities, such as diabetes mellitus, cardiovascular and cerebrovascular diseases, and structural lung diseases. Pulmonary infection in this vulnerable group frequently exacerbates these underlying conditions, resulting in poor clinical outcomes and even mortality (Hua et al., 2021; Zhou et al., 2023). Indwelling urinary catheters — the most commonly used healthcare-associated catheters — and catheter-related urinary tract infection (UTI) are highly prevalent in elderly patients, with previous studies suggesting potential associations with adverse prognoses (Alemu et al., 2020; Shen et al., 2023). In view of this clinical gap, the present study focused on the specific elderly cohort to characterize the clinical use of IUC and systematically clarify the respective impacts of IUC and UTI occurrence on the short-term prognosis of elderly patients with PI.

Finally, a total of 700 newly admitted elderly patients with PI were included in this study, all aged ≥65 years, with a substantial proportion of “super-elderly” individuals (≥85 years). Consistent with the well-documented vulnerability of this age group, most patients had multiple comorbidities, and the overall 30-day mortality rate was high (17.3%, 121/700). Notably, 42.1% (295/700) of patients underwent IUC within 72 hours of admission—a rate significantly higher than that reported in the general hospitalized population in previous studies (Czwikla et al., 2023). Among the entire cohort, 18.9% (132/700) had both IUCs and UTIs; however, the retrospective design of this study precluded definitive determination of whether these UTIs were catheter-associated.

Baseline comparisons (**Table 1**) revealed that IUC was associated with more severe disease phenotypes: patients with IUCs had a higher proportion of CURB-65 scores >2 (73.7% vs. 37.2%), higher rates of Respiratory Intensive Care Unit (RICU) admission (49.2% vs. 21.7%), and elevated 30-day mortality (28.1% vs. 9.4%) compared to those without IUCs. These unadjusted data initially suggested a significant association between IUC and poor prognosis (30-day mortality) in elderly PI patients.

To disentangle the independent effects of IUCs and UTIs on mortality, Kaplan-Meier (KM) survival analysis was performed (**Figure 1**). The results confirmed that IUC was significantly associated with reduced 30-day survival (P < 0.05), whereas UTI status had no significant impact on 30-day mortality (P > 0.05). This finding aligns with a prior study by Yamazoe et al. (2019), which demonstrated that UTI could not independently predict mortality in healthcare-associated pneumonia elders; however, their research did not stratify patients by IUC exposure and failed to separate the prognostic effect of catheters from UTI. Our finding is also consistent with a prior study by Oka et al. (2017), which confirmed pyuria had no adverse impact on pneumonia prognosis, yet it did not analyze the direct prognostic role of IUC. Most existing studies on IUC and mortality recruited heterogeneous mixed ICU cohorts with various primary diseases, while our research is the first retrospective cohort specifically focusing on elderly patients with PI to distinguish the distinct prognostic effects of IUC and UTI, filling the research gap mentioned in the Introduction section. Our findings further support the notion that IUCs—rather than UTIs—are the primary driver of poor prognosis in this population.

However, our results also indicated that patients with IUC appeared to have more severe illness and had a higher proportion of RICU admissions (**Table 1**), and these markers of severe illness represent clear risk factors for poor prognosis. Therefore, we applied Cox regression to analyze the predictive value of IUC for poor prognosis. As shown in **Table 2**, the univariate analysis identified IUC as a strong risk factor for 30-day mortality (HR = 2.7, 95% CI: 1.8–3.9, P < 0.001). When UTI was included as an adjustment variable (Model 1), IUCs remained an independent risk factor (HR = 2.8, 95% CI: 1.9–4.2, P < 0.001). However, after further adjusting for urinary tract infection, CURB-65 score ≥2, RICU admission and female gender (Model 2), IUC no longer qualified as an independent risk factor, despite a 40% increased mortality risk (HR = 1.4, 95% CI: 0.9–2.1, P = 0.149). After adding mechanical ventilation into the fully adjusted model (Model 3 in **Table 2**), the predictive effect of IUC on 30-day mortality was further weakened (HR = 1.3, 95% CI: 0.8–2.0, P = 0.246).

Therefore, these findings above suggest that the association between IUCs and poor prognosis in elderly PI patients may be mediated by the severity of underlying disease. IUC is often a marker of more severe illness — evidenced by higher CURB-65 scores, higher RICU admission rates, and higher mechanical ventilation rates — which directly contributes to increased mortality. Of course, IUCs might also indirectly worsen outcomes by causing catheter-related complications. Some studies report that long-term catheterization (over 10 days) is associated with up to a 93% incidence of bacteriuria (Chenoweth and Saint, 2016; Chenoweth, 2021), and it significantly raised the risk of multidrug-resistant bacterial infections (Yang et al., 2022), which could make PI worse or trigger secondary infections. From the perspective that IUC can increase urinary tract infections, the distribution characteristics of pathogens causing UTIs are also very important for clinical treatment and prognosis. Unfortunately, the retrospective nature of this study prevented us from confirming a causal link between IUCs and UTI development, highlighting the need for prospective studies to clarify this mechanism. Meanwhile, since the positive rate of urine cultures in our cohort was relatively low, we were unable to obtain detailed data on the proportions of each pathogen. However, existing systematic studies widely acknowledged the conclusion that the distribution of pathogens in urinary tract infections was highly homogeneous. To enhance the translational value of our study results, we added the epidemiological characteristics of urinary tract infection pathogens and their corresponding clinical significance in this section, as follows:

Extensive authoritative epidemiological studies and clinical guidelines have consistently demonstrated that due to the anatomical proximity of the urinary tract, genital tract, and rectum, Gram-negative bacilli dominate the pathogen spectrum of both catheter-associated and non-catheter-associated nosocomial UTIs, with Escherichia coli serving as the most prevalent uropathogen in hospitalized elderly populations (Genao and Buhr, 2012; Alebachew et al., 2025). Accumulated clinical epidemiological evidence confirms that the pathogenic spectrum of hospital-acquired UTI in elderly and critically ill patients is highly stable and homogeneous across different medical centers (Alebachew et al., 2025). Gram-negative bacilli constitute the predominant pathogens, among which Escherichia coli accounts for 40%–60% of all culture-positive isolates, followed by Klebsiella pneumoniae and Pseudomonas aeruginosa (Genao and Buhr, 2012; Pathi et al., 2025). For immunocompromised elderly patients and those with prolonged IUC, Gram-positive pathogens (primarily Enterococcus faecalis) and Candida fungi are frequently detected as opportunistic infectious pathogens, constituting an important supplementary component of nosocomial UTI pathogens (Genao and Buhr, 2012). This uniform microbial distribution feature indicates that the flora characteristics of UTI in elderly hospitalized patients are highly consistent in real-world clinical settings, which provides a solid epidemiological basis for standardized empirical anti-infective treatment.

Besides the conclusion mentioned above that IUC can indicate the severity of illness in elderly patients with lung infections, the results also underscore the importance of prudent IUC use in elderly patients with PI from a clinical perspective. The Guideline for Prevention of Catheter-Associated Urinary Tract Infections explicitly recommends IUCs only for short-term management of conditions such as acute urinary retention and perioperative monitoring, and discourages their routine use for long-term urinary assistance in elderly patients (Gould et al., 2010). However, studies indicate that a large proportion of IUCs lack clear medical indications, with overuse particularly prevalent among bedridden elderly patients. Our data—showing that 42.1% of elderly PI patients in a respiratory ward had IUCs—further confirms this issue. To improve patient safety, enhance prognosis, and reduce healthcare costs, several strategies should be implemented to optimize IUC use: (1) Strictly adhere to IUC indications, avoiding use for “convenience of care” rather than medical necessity; (2) Implement daily assessment of catheter necessity and prompt removal when no longer indicated to minimize catheterization duration; (3) Promote alternative urinary management solutions, such as absorbent pads, external urinary catheters, and incontinence underwear, to reduce reliance on invasive IUCs.

In conclusion, our stratified survival and regression analyses clarified that IUC, instead of UTI, serves as the key predictive factor for adverse short-term prognosis in this patient population. IUC exhibited significant independent prognostic value for elevated 30-day mortality when only UTI was adjusted for confounding. However, after comprehensive adjustment for multiple clinically critical confounding factors, including disease severity (urinary tract infection, CURB-65 score ≥2, RICU admission, female gender and mechanical ventilation), IUC no longer remained an independent risk factor for mortality. Notably, IUC still conferred a 30% increase in 30-day mortality risk among elderly patients with pulmonary infection, with a marginal statistical trend toward poor prognosis. Collectively, these findings indicate that the adverse prognostic association of IUC is largely mediated by aggravated baseline disease severity, rather than being an independent pathogenic factor, suggesting that elderly pulmonary infection patients with IUC deserve intensified clinical attention and refined standardized management to further improve their short-term prognosis.

## Limitations

First, since this study was a retrospective study, complete and accurate electronic medical records of IUC indications and exact catheter indwelling duration were unavailable for all patients. 48 of 295 catheterized patients had no clear documented catheterization indications, and stratified analysis and statistical adjustment based on catheterization duration could not be performed, which may bring potential residual confounding bias, though this bias cannot reverse our core research conclusion. We propose that future prospective studies should collect complete dynamic catheterization records to incorporate IUC indications and indwelling duration as comprehensive adjustment covariates to further validate our findings. Second, only a tiny number of patients in this cohort combined mild subclinical bloodstream infection (BSI). Due to incomplete microbial dynamic data in retrospective medical records, we cannot reliably distinguish whether BSI originated from hematogenous dissemination of UTI or independent concurrent infection caused by severe pulmonary infection and immune decline, so we did not carry out statistical analysis on BSI to avoid biased clinical inference. We plan to design prospective studies with complete microbial monitoring data to further explore the correlation between UTI and BSI in elderly pneumonia patients. Third, this is a single-center retrospective study focusing exclusively on elderly patients with pulmonary infection admitted to respiratory wards and RICU, which may limit the generalizability of our conclusions. Multi-center large-sample prospective studies covering various hospitalized elderly populations will be carried out in follow-up research to externally verify our results. Lastly, the retrospective nature precluded definitive confirmation of a causal relationship between indwelling urinary catheter (IUC) placement and urinary tract infection (UTI) development. As such, this study could not verify whether IUCs exacerbate prognosis indirectly by inducing UTIs. Resolving these uncertainties will require well-designed prospective studies in future that can systematically track catheterization status, UTI onset, and clinical outcomes over time.

## Data Availability

The raw data supporting the conclusions of this article will be made available by the authors, without undue reservation.
